# Tethered agonist activated ADGRF1 structure and signalling analysis reveal basis for G protein coupling

**DOI:** 10.1038/s41467-023-38083-7

**Published:** 2023-04-29

**Authors:** Daniel T. D. Jones, Andrew N. Dates, Shaun D. Rawson, Maggie M. Burruss, Colin H. Lipper, Stephen C. Blacklow

**Affiliations:** 1 Department of Biological Chemistry and Molecular Pharmacology, Blavatnik Institute, Harvard Medical School, Boston, MA 02115 USA; 2 Department of Cancer Biology, Dana Farber Cancer Institute, Boston, MA 02215 USA

**Keywords:** Electron microscopy, Biochemistry

## Abstract

Adhesion G Protein Coupled Receptors (aGPCRs) have evolved an activation mechanism to translate extracellular force into liberation of a tethered agonist (TA) to effect cell signalling. We report here that ADGRF1 can signal through all major G protein classes and identify the structural basis for a previously reported Gα_q_ preference by cryo-EM. Our structure shows that Gα_q_ preference in ADGRF1 may derive from tighter packing at the conserved F569 of the TA, altering contacts between TM helix I and VII, with a concurrent rearrangement of TM helix VII and helix VIII at the site of Gα recruitment. Mutational studies of the interface and of contact residues within the 7TM domain identify residues critical for signalling, and suggest that Gα_s_ signalling is more sensitive to mutation of TA or binding site residues than Gα_q_. Our work advances the detailed molecular understanding of aGPCR TA activation, identifying features that potentially explain preferential signal modulation.

## Abstract (web summary)

ADGRF1 is an adhesion GPCR that signals when a bound ligand induces liberation of an intramolecular tethered agonist. Here, the authors show that ADGRF1 can signal though all major G protein classes and identify a structural basis for Gq selectivity.

## Introduction

Adhesion G protein coupled receptors (aGPCRs) are the second largest class of GPCRs, constituting 33 members across 9 subfamilies^1^. aGPCRs control a multitude of cellular processes involved in organ development and tissue homeostasis, responding to external stimuli that sense extracellular physical forces, such as binding to protein ligands presented on cells, components of extracellular matrix and shear flow^2^. Mutations in aGPCRs are genetically responsible for human developmental disorders, such as ADGRV1 in Usher syndrome^3^, and EMR2 in vibratory urticaria^4^.

aGPCRs have a unique modular architecture, with an N-terminal ectodomain containing extracellular adhesive modules, followed by a G protein coupled receptor autoinducing (GAIN) domain linked to a seven-transmembrane (7TM) bundle that relays extracellular events to the cell^5,6^. A distinguishing hallmark of aGPCRs is that the GAIN domain undergoes autoproteolysis at a short hairpin turn between the final two beta-strands, separating the aGPCR into non-covalently attached N-terminal and C-terminal fragments, named NTF and CTF respectively^7,8^. Current models for aGPCR activation posit that mechanical force applied to the adhesive modules in aGPCRs induces dissociation of the NTF from the CTF^9,10^, enabling the newly liberated N-terminal end of the CTF to act as an intramolecular agonist to the 7TM domain. Intramolecular ligation of this tethered agonist (TA) to the 7TM domain relays the initial extracellular cue to the cell through G protein coupling or beta-arrestin activity.

The first structure of an aGPCR 7TM domain was solved for ADGRG3 (GPR97) bound to glucocorticoids in the orthosteric pocket, revealing key residues involved in ligand recognition, conformational switches important for receptor activation, and residues for coupling to G protein^11^. Cryo-EM structures were then reported for seven different aGPCR family members activated by their TAs, including representative GPCR-G protein complexes for all major families of Gα^12–16^. These studies showed that the TA acts an intramolecular ligand for the 7TM domain and identified a canonical binding pose for a conserved hydrophobic interaction motif (TØFØØLM) of the TA in the GPCR orthosteric site.

ADGRF1 (GPR110) is an aGPCR belonging to aGPCR subfamily VI^1,17^. ADGRF1 was identified as an oncogene overexpressed in lung and prostate cancer^18^, and has been implicated in synaptamide dependent neural outgrowth and repair^19,20^, though this activity remains controversial^21^. ADGRF1 was used as a prototypical aGPCR to determine that membranes expressing ADGRF1 treated with urea at high concentration induced shedding of the NTF, liberating the TA to activate the 7TM and recruit Gα_q_ preferentially over other Gα proteins^10^. Further work has also showed that mouse ADGRF1 can be activated by synthetic TA peptides when added in trans, signalling through both Gα_s_ and Gα_q_^22^.

Recent structures of the TA-engaged state of the ADGRF1 CTF coupled to either miniGα_s_ or miniGα_i_ also identified a lysophosphatidylcholine (LPC) lipid bound at the intracellular convergence of TM helices II, III and IV^15^. Interestingly, fragments of ADGRF1 expressed with the GAIN domain included also yielded TA-engaged structures of ADGRF1 coupled to miniG but lacked density for the GAIN domain, suggesting that the CTF may have dissociated from the NTF portion of the protein during purification. Additionally, using a HiBiT tethering approach, structures of ADGRF1 have been solved bound to a representative miniG of each G protein class, raising the possibility that ADGRF1 is a promiscuous G protein coupler^16^, but the HiBiT tethering approach artificially recruits receptors to G proteins, and thus the ADGRF1 G protein signalling profile remains unclear.

Here, we extensively characterise ADGRF1 G protein signalling. First, using transcription factor assays and deletion complementation by transient transfection in HEK293T cells lacking certain G proteins, we deconvolute the G proteins responsible for CRE, NFAT-RE and SRF signalling in cells. As a more direct measurement of G protein coupling, we show using TRUPATH G protein biosensors, that ADGRF1 can convert a representative member of each G protein class to an active state. To understand the molecular events driving TA activation of ADGRF1 and how its structure determines Gα class preference, we determined a cryo-EM structure of TA-activated ADGRF1 coupled to miniGα_s/q_-β_1_γ_2_, stabilised by Nb35. Combining structural insights with extensive functional studies, we identify key residues of the orthosteric binding site that are essential for Gα_s_ signalling but dispensable for Gα_q_ signalling. Overall, our work complements other recent aGPCR structures^12–16^ and extends them by visualizing ADGRF1 in complex with its most relevant G protein partner in a non-tethered complex, elucidating a functional and structural landscape by which ADGRF1 is activated and uncovering how its structure determines Gα class preference. Furthermore, the mechanistic insights determined here regarding TA engagement to strong and weakly associated G proteins offers valuable insight into how TA mediated activation of aGPCRs may be functionally modulated.

## Results

### ADGRF1 signals through all G protein classes

**Fig. 1:**
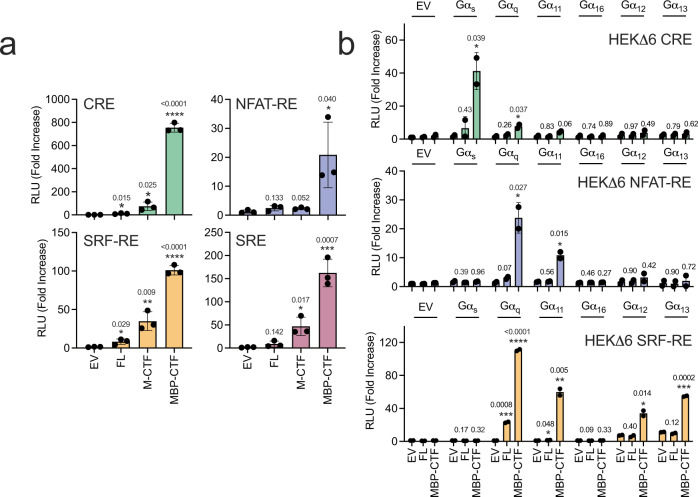
Analysis of G protein coupling of ADGRF1 using CRE, NFAT-RE and SRF-RE reporters in parental and HEKΔ6 knockout cells. **a** Transcription factor reporter assay responses for ADGRF1 FL, M-CTF or MBP-CTF relative to empty vector (EV) for CRE (top left), NFAT-RE (top right), SRF-RE (bottom left) and SRE (bottom right). Cells were transfected with 30 ng receptor DNA/well for SRE and SRF-RE, and 10 ng DNA/well for CRE and NFAT-RE. Data are normalized to empty vector as relative luminescence units (RLU). Data are presented as mean ± s.d. of three biological replicates. Each point represents the mean value of three technical replicates. Student’s two-tailed T-test (*P < 0.05, **P < 0.01, ***P < 0.001, and ****P < 0.0001) was used to compare the means between biological samples to each reporter’s empty vector control. **b** Gα complementation assay. Activity of the CRE, NFAT-RE and SRF-RE reporters were measured in HEKΔ6 cells transfected with a combination of EV, ADGRF1 FL or MBP-CTF, and either EV or one of several different Gα subunits. All Gα were transiently transfected at 20 ng DNA per well, except for Gα_s_ in the CRE assay which was given at 1 ng, and Gα_12/13_ which were given at 0.3 ng in the SRF-RE assay. Values are reported as the ratio of the raw luminescence values of firefly and renilla luciferases normalized to the matched Gα empty vector (receptor) control. Data are presented as mean ± s.d. of two biological replicates. Each point represents the mean value of three technical replicates. In all panels, Student’s two-tailed T-test (*P < 0.05, **P < 0.01, ***P < 0.001, and ****P < 0.0001) was used to compare the means between biological samples to each reporter’s empty vector control.

We recently developed a platform for analysis of the signalling activity of aGPCRs, in which the CTF of interest is expressed as a protein fusion with an N-terminal IL2 signal sequence followed by maltose binding protein (MBP) and a tobacco etch virus cleavage site (denoted MBP-CTF)^23^ (Supplementary Fig. 1). A FLAG tag is also appended to the C-terminal end of the protein. Using this approach to measure signalling activity in transcription factor reporter assays, we observed transcriptional responses with both CRE and NFAT-RE reporters, consistent with previous studies^15,22^, as well as with SRE and SRF-RE reporters (Fig. 1a).

To further evaluate the coupling spectrum of ADGRF1, and to investigate crosstalk of G proteins as inducers of the various transcriptional responses tested, we used HEK293T cells with deletion of six Gα subunits (Gα_s_ short, Gα_s_ long, Gα_q_, Gα_11_, Gα_12_ and Gα_13_; gift from A. Inoue, Tohoku university, Japan)^24^, and performed deletion complementation by transient transfection to determine which Gα subunits support ADGRF1 signalling in the CRE, NFAT-RE and SRF-RE reporter assays (Fig. 1b).

In CRE reporter assays with these cells, we observed a significant increase in basal signalling only upon transfection of cells with 20 ng of the Gα_s_ subunit; all other Gα subunits could be dosed at 20 ng without an increase in basal signalling. We therefore titrated the dose of Gα_s_ DNA and found that 1 ng of DNA was the highest DNA dose that did not raise basal signalling compared to empty vector control. When CRE reporter activity was assessed, a strong, significant increase in activity was observed when the MBP-CTF form of ADGRF1 was co-transfected with 1 ng Gα_s_, whereas a much smaller (but significant) increase was observed with 20 ng Gα_q_ and MBP-CTF (Fig. 1b, top). These data show that CRE reporter activity induced by the ADGRF1 CTF results primarily from Gα_s_ coupling.

In NFAT-RE reporter assays all Gα subunits could be transfected at 20 ng DNA per well with no increase in basal signalling activity. When ADGRF1 MBP-CTF was co-transfected with either Gα_q_ or Gα_11_, a robust, significant increase in signalling is observed, whereas no increase in basal signalling is seen upon co-transfection of the other Gα subunits (Fig. 1b, middle). Given that only co-transfection of Gα_q_ or Gα_11_ with MBP-CTF elicits a response, the NFAT-RE reporter shows fidelity to Gα_q_ signalling,

In SRF-RE reporter assays, a significant increase in basal signalling is observed when cells are transfected with 20 ng of plasmid encoding Gα_12_ or Gα_13_, whereas plasmids encoding all other Gα subunits could be dosed at 20 ng without an increase in basal activity. Again, we titrated Gα_12_ and Gα_13_ DNA to identify the highest plasmid dose that did not result in an increase of basal signalling, which was 0.3 ng of DNA for each. When the optimized dose of each Gα subunit was co-transfected with MBP-CTF, significant increases in SRF-RE signalling were observed for Gα_q_, Gα_11_, Gα_12_, and Gα_13,_ and a small but significant increase is also seen with ADGRF1 FL in the presence of Gα_q_ (Fig. 1b, bottom). SRF-RE therefore appears to report both on Gα_q/11_ and Gα_12/13_ signalling based on the responses seen upon complementation with these G proteins.

**Fig. 2:**
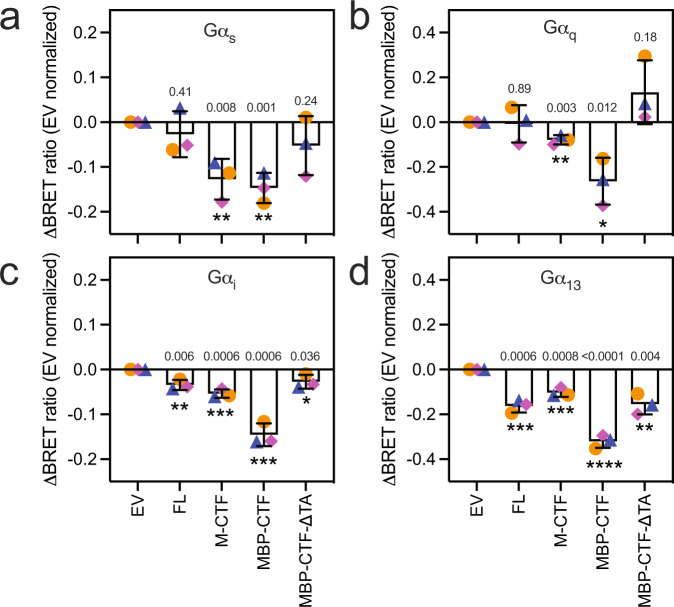
Evaluating G protein coupling of TA-engaged ADGRF1 for all G protein classes using the TRUPATH BRET assay. G protein activation assays, referenced to empty vector (EV), were performed for ADGRF1 FL, M-CTF, MBP-CTF, or MBP-ΔTA-CTF using TRUPATH Gα_s_ (**a**), Gα_q_ (**b**), Gα_i_ (**c**), and Gα_13_ (**d**) systems. Cells were transfected with 20 ng DNA/well of each receptor variant, and 80 ng DNA/well of the TRUPATH plasmid. BRET ratios are normalized to the empty vector control of each receptor condition paired with each tested G protein (**a–d**). Data are presented as mean ± s.d. of three biological replicates. Symbols and colours depict experiments from the same biological replicate (orange circles, blue triangles, magenta diamonds). Student’s two-tailed T-test was used to compare the means between biological samples to each reporter’s empty vector control (*P < 0.05, **P < 0.01, ***P < 0.001, and ****P < 0.0001).

To evaluate ADGRF1 G protein signalling promiscuity further, we used the TRUPATH GPCR proximal BRET assay to interrogate G protein heterotrimer activation in cells^25^. In this assay, BRET from a Gα-*Renilla-*Luc8 fusion protein to Gγ_2_-GFP decreases when G protein turnover takes place and either shows minimal change or becomes elevated when the G protein pool remains in an inactive or complexed heterotrimeric state. We measured the BRET ratio in the TRUPATH assay for cells transfected with empty vector, full length ADGRF1, M-CTF, MBP-CTF, or MBP-ΔTA-CTF, from which the seven-residue sequence constituting the tethered agonist (TSFSILM) was deleted. We observed that G proteins representative of all major classes show a significant reduction in the BRET ratio for M-CTF and MBP-CTF relative to empty vector control (Fig. 2). We note that the BRET ratio decreases for all forms of ADGRF1 tested when Gα_13_ is the G protein partner, including the MBP-ΔTA-CTF protein that lacks the tethered agonist, suggesting some tethered agonist independent turnover of Gα_13_ is detectable for ADGRF1 in this assay. Nevertheless, the significance of BRET reduction when the tethered agonist is present is greater than in its absence, indicating a TA-dependent increase in response for Gα_13_ as well. Taken together with the transcription factor reporter assays, the TRUPATH results indicate that ADGRF1 has the capacity to transduce signals through Gα_s_, Gα_q_, Gα_i_ or Gα_12/13_, and thus appears to be the first of the class B1 or B2 GPCRs shown to have the capability to signal through all G protein classes^26^.

### Protein engineering of ADGRF1 and Cryo-EM structure determination

To obtain an active state ADGRF1 G protein complex amenable for structural and functional investigation, we probed complex assembly with a suite of miniGα proteins^27,28^, using a NanoBiT recruitment assay^29,30^. Unlike HiBiT, which has a strong intrinsic affinity for LgBiT (~700 pM) and therefore forces the tethering of two proteins^31^, The SmBiT sequence used here has a very weak intrinsic affinity for LgBiT (~190 μM), and requires a biologically relevant binding interface between a GPCR and Gα protein for formation of complexes^31^. We found that LgBiT-miniGα_s_, miniGα_s/q_ and miniGα_o_ were all recruited to C-terminal SmBiT-tagged ADGRF1-CTF as judged by increased luminescence upon SmBiT-LgBiT complementation, whereas matched CΔ5 control forms of these mini G proteins and LgBiT-miniGα_12_ were not recruited (Supplementary Fig. 2a). To assess tractibility for structure determination, we co-expressed ADGRF1 with these miniG constructs in Expi293T suspension cells (ThermoFisher, A14527), and also observed a similar pattern of Gα recruitment to ADGRF1 (Supplementary Fig. 2b, left). When we solubilised cells with mild detergent supplemented with cholesterol in the presence of apyrase, the only complex that retained luminescence under these lysis conditions was the combination of SmBiT-tagged ADGRF1-CTF with LgBiT-miniGα_s/q_ (Supplementary Fig. 2b, right). In addition, only miniGα_s/q_ specifically purified with ADGRF1-CTF using anti-FLAG M2 magnetic beads (Supplementary Fig. 2c), indicating that ADGRF1-Gα complex stability is greater for miniGα_s/q_ than for miniGα_s_ or miniGα_o_. Furthermore, miniGα_s/q_ co-expression with ADGRF1-CTF appears to increase the amount of receptor purified, suggesting that the miniGα_s/q_ may improve receptor expression by facilitating the folding of the receptor, increasing its stability, or by blocking toxic signalling through endogenous signalling pathways.

### Structure of the ADGRF1-miniGα_s/q_ heterotrimeric G protein complex with the Nb35 nanobody

**Fig. 3:**
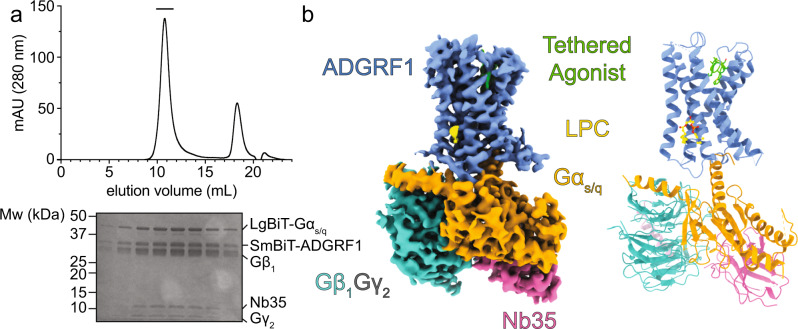
ADGRF1-miniGα_s/q_-Nb35 complex purification and cryo-EM structure. **a** ADGRF1 miniGα_s/q_-Gβ_1_γ_2_-Nb35 purification. Top: elution profile of the complex after size-exclusion chromatography as a final purification step. Bottom: peak fractions (bar above chromatogram) analyzed by SDS-PAGE. **b** Structure of the ADGRF1-Gα_s/q_-β_1_γ_2_-Nb35 complex. Left: cryo-EM map of the ADGRF1 complex. ADGRF1 (light blue), the TA (green), Gα_s/q_ (orange), Gβ_1_ (teal), Gγ_2_ (grey), and Nb35 (pink) are coloured as indicated. The density threshold was set at five standard deviations from the mean. Right: cartoon rendering of the structure, with residue side chains of the tethered agonist rendered as sticks. The density threshold was set at three standard deviations from the mean for the TA. The LPC lipid (yellow) molecule is shown with a ball-and-stick model.

To elucidate the basis for selective recruitment of Gα_s/q_, we complexed ADGRF1 CTF with a miniGα_s/q_ heterotrimeric G protein complex and the Nb35 nanobody, purified the complex by size-exclusion chromatography (Fig. 3a), and determined its structure by cryo-EM to 3.4 Å resolution (Supplementary Figs. 3, 4). The cryo-EM map was of sufficient resolution to permit the building of an atomic model for the receptor, G protein complex, and nanobody (Fig. 3b, Supplementary Fig. 5). The cryo-EM map in the orthosteric pocket on the extracellular face of the receptor permitted unambiguous modelling of the TA (Supplementary Fig. 5), which adopts a short alpha-helical motif with a loop capping the TA en route to TM1, akin to other resolved TA structures of aGPCRs^12–15^. The polypeptide backbone of the 7TM domain of ADGRF1 bound to miniGα_s/q_ has an RMSD of 0.6 Å when aligned to structures of the 7TM domain of ADGRF1 bound to either miniGα_s_ (PDB 7WU3) or miniGα_i_ (PDB 7WU4)^15^ highlighting that the overall organization of these structures is highly similar despite their different G protein partners. An LPC lipid is present at the intracellular convergence of TM helices II, III, and IV, as also seen in ADGRF1 structures bound to miniGα_s_ or miniGα_i_^15^. The acyl chain is similarly positioned in our structure, whereas the headgroup has the phosphate embedded deeper in the detergent micelle (Fig. 3b, Supplementary Fig. 5).

### Structural differences between the ADGRF1 miniGα_s/q_ and ADGRF1 miniGα_s_ complexes

MiniGα_s/q_ was engineered as a chimera with six key Gα_q_ residues on the C-terminal α5 helix that distinguish Gα_q_ coupling over Gα_s_, even though the bulk of the chimera is derived from miniGα_s_^27^. Because we observe a preference for miniGα_s/q_ recruitment to the CTF of ADGRF1, consistent with the previously observed preference for Gα_q_ in GTPγS activity assays^10^, the preference for Gα_q_ is driven largely by the differences in its C-terminal α5 helix.

**Fig. 4:**
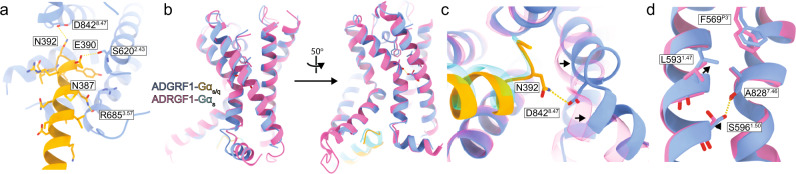
The ADGRF1-miniGα_s/q_ interface and comparison to ADGRF1-miniGα_s_. **a** Close-up of the ADGRF1 (blue) interface with Gα_s/q_ (orange) along the Cα5 helix of Gα_s/q_. Hydrogen bonding interactions between the receptor and Gα are indicated with yellow dashed lines. **b** Comparison of the ADGRF1 (blue)—Gα_s/q_ (orange) complex with the ADGRF1 (pink)—miniGα_s_ (cyan) complex, aligned by least-squares superposition of the ADGRF1 subunits. **c** Zoomed in view focusing on D842^8.47^ in the turn connecting helices VII and VIII. The hydrogen bond between N392 and the carboxylate of D842^8.47^ is shown with a yellow dotted line. **d** Zoomed in view focusing on residues L593^1.47^ and S596^1.50^. Arrows indicate positional shifts of these residues in the miniGα_s/q_ structure relative to their positions in miniGα_s_. The hydrogen bond between S596^1.50^ and the backbone carbonyl of A828^7.46^ is shown with a yellow dotted line.

At the ADGRF1 G protein interface we observe a canonical pose of the C-terminal α5 helix at the open cavity at the intracellular opening of TA activated ADGRF1 (Figs. 3b, 4a). Three hydrogen bond interactions appear to stabilise the ADGRF1-Gα_s/q_ interface in our structure. The N387 amide side chain nitrogen of miniGα_s/q_ approaches within hydrogen bonding distance of the backbone carbonyl of ADGRF1 R685^3.57^, E390 carboxylate approaches within hydrogen bonding distance of the hydroxyl group of S620^2.43^ and the amide side chain nitrogen of N392 approaches within hydrogen bonding distance of D842^8.47^ side chain carboxyl group (Fig. 4a).

The analogous residues of miniGα_s_ to N387, E390, N392 in miniGα_s/q_ are H387, Q390 and E392, which do not form the same hydrogen bonding arrangement as in the Gα_s/q_ complex. In the Gα_s_ complex, H387 does not make any polar interactions, Q390 approaches within hydrogen bonding distance of the backbone amide nitrogen of S617^2.40^ and the S620^2.43^ hydroxyl group and E392 approaches within hydrogen bonding distance of the backbone amine of S843^8.48^ (Supplementary Fig. 6a). Given the close proximity of E392 to D842^8.47^, It is likely that charge repulsion destabilises the miniGα_s_-bound state by comparison to the Gα_q_-bound state. These interactions are associated with a shift in the positioning of the segment linking TM helix VII to helix VIII relative to the Gα subunit, with the Cα of D392 in the α5 helix of miniGα_s_ situated 1.9 Å closer to the Cα of ADGRF1 S843^8.48^ (Fig. 4c). The arrangement seen in the miniGα_s_ complex is also present in the miniGα_i_ structure. Superposition of helices I and VII of miniGα_s/q_ on miniGα_s_ shows a 3.2 Å displacement of the L384 Cα miniGα_s/q_ relative to that of N384 of miniGα_s_ (Supplementary Fig. 6b).

At the orthosteric site, we also observe a 1.2 Å displacement of L593^1.47^ towards F569^P3'^ in the ADGRF1 complex with miniGα_s/q_, compared with the miniGα_s_ and miniGα_i_ complexes (Fig. 4d). This inward movement leads to a rearrangement of the TM I alpha helix, allowing the conserved S596^1.50^ hydroxyl to approach within hydrogen bond distance of the backbone carbonyl of A828^7.48^ at the kink in the TM VII helix. This interaction of S569^1.50^ with TM helix VII is not observed in either the miniGα_s_ or miniGα_i_ structures.

A structure of ADGRF1 bound to miniGα_s/q_ was also recently solved using the HiBiT tethering approach^16^. When the 7TM domain of our structure is aligned with the ADGRF1-miniGα_s/q_ tethered structure (PDB 7WXU), the RMSD over all atoms in the 7TM domain is 0.8 Å. However, the position of I839^7.58^ is subtly different in the tethered structure, which affects the relative positions of TM helix VII and helix VIII to TM helix I (Supplementary Fig. 7). As a result the miniGα_s/q_ tethered structure more closely resembles that of the untethered miniGα_s_ structure (PDB 7WU3)^15^. The side-chain interactions of N387, E390 and N392 of miniGα_s/q_ with R685^3.57^, S620^2.43^ and D842^8.47^, respectively (described above) are also observed in the tethered structure^16^. The tethered structure additionally reports the presence of interactions between the sidechains of miniGα_s/q_ residues D381 and Q385 with the guanidinium group of R771^5.63^, as well as an interaction between V394 at the C-terminus with the side chain of K791^6.40^. In our structure, there is not appreciable density for R771^5.63^, suggesting flexibility in this region. Similarly, the side chain position of K791^6.40^ is not well defined in our electron density map, suggesting that the position of this side chain is also flexible. Further analysis of coupling contacts to miniGα_i_, miniGα_12_ and miniGα_13_ responsible for ADGRF1 G protein coupling promiscuity and comparisons among these structures, determined using the tethering approach, are available elsewhere^16^.

### Distinguishing preferential Gα_q_ over Gα_s_ tethered agonism at the orthosteric site

**Fig. 5:**
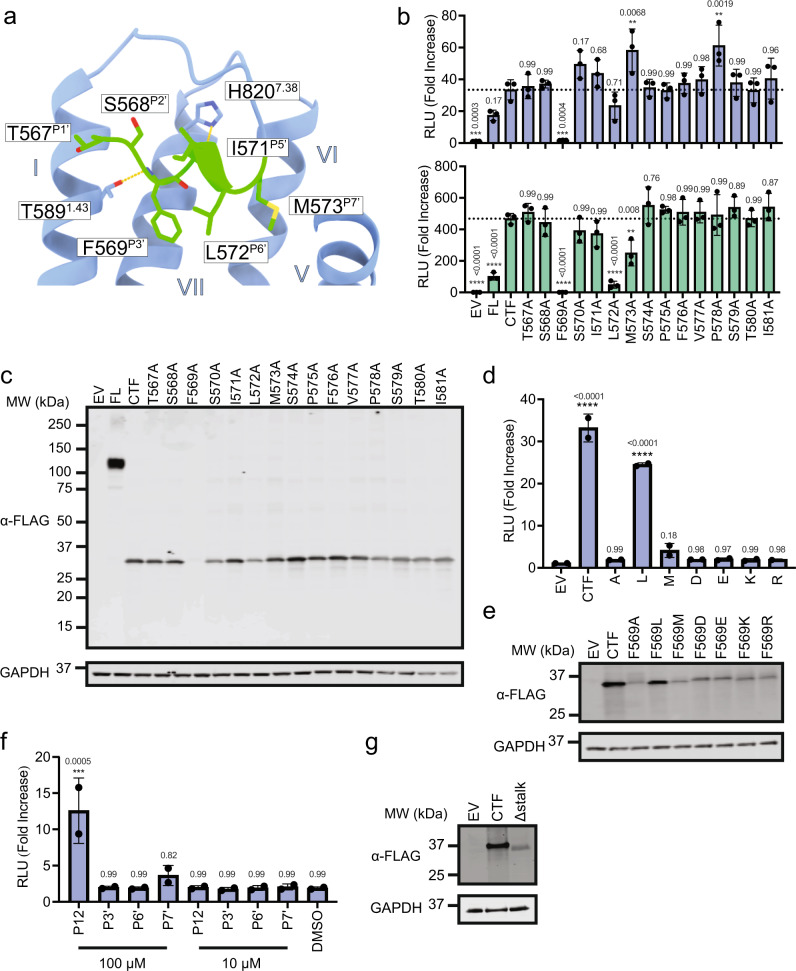
Tethered agonism signalling determinants through NFAT-RE and CRE. **a** Pose of the TA in the orthosteric site. TA sidechain atoms and main-chain atoms making polar contacts are shown as sticks. Sidechains of 7TM residues making polar contacts (yellow dashes) to the TA are also shown. TA superscript labels refer to the TA position relative to the GPS autoproteolysis site. Receptor residue superscript labels refer to Wootten numbering. **b** Scanning alanine mutagenesis of the TA. NFAT-RE and CRE reporter data were acquired using ADGRF1 CTF as a reference sequence. Cells were transfected with 20 ng of receptor DNA/well. Data are normalized to empty vector and reported as relative luminescence units (RLU). The mean for the CTF is indicated as a horizontal dotted line. **c** α-FLAG Western blot comparing steady-state amounts of wild-type and mutated ADGRF1 CTF proteins tested in panel b. An α-GAPDH blot was used as a loading control. Blot shown is representative of three independent biological repeats. **d** NFAT-RE signalling data and (**e**) α-FLAG Western blot of wild-type and P3' mutated forms (L, M, R, K, D and E) of the ADGRF1 CTF. An α-GAPDH blot was used as a loading control. Blot shown is representative of two independent biological repeats. **f** NFAT-RE reporter gene activity of the ADGRF1 CTFΔstalk protein stimulated by *trans* addition of a 12-residue TA peptide (P12). The response to the wild-type peptide or to peptides with alanine mutations at the P3', P6' or P7' positions are shown at doses of 10 or 100 μM. **g** α-FLAG Western blot comparing steady-state amounts of wild-type and Δstalk ADGRF1 CTF proteins tested in **f**. An α-GAPDH blot was used as a loading control. Blot shown is representative of two independent biological repeats. For panel (**b**), data are presented as mean ± s.d. of three biological replicates. Each point represents the mean value of three technical replicates. For panels **d** and **f** data are presented as mean ± s.d. of two biological replicates. Each point represents the mean value of three technical replicates. One-way analysis of variance (ANOVA) was used with Dunnett’s multiple-comparison post-hoc test to compare the difference between CTF to all other samples individually in **b** and **d**, and to DMSO in **f** (**P < 0.01;***P < 0.001;****P < 0.0001).

Previous work using truncations and alanine substitutions at the P3' and P7' positions identified the functional importance of hydrophobic residues in TA-mediated signalling^10^. Because ADGRF1 shows preference for Gα_q_ over other Gα partners^10^, we investigated here whether Gα_q_ and Gα_s_ signalling exhibited differential sensitivity to alanine mutants of the receptor stalk residues (Fig. 5a), using NFAT-RE and CRE reporter gene assays for Gα_q_ and Gα_s_ coupling, respectively (Fig. 5b). Positions 574–581, corresponding to P8'-P15', were largely unaffected by mutation to alanine in NFAT-RE and CRE assays, indicating that this region is not directly important for TA dependent agonism, primarily functioning as a linker connecting the TA to TM1 of ADGRF1.

As in a previous analysis of the F569^P3'^A and M573^P7'^A mutations^10^, alanine substitutions at F569^P3'^ and M573^P7'^ of the TA affected TA-dependent signalling, as did an alanine substitution at L572^P6'^. The L572^P6'^A and M573^P7'^A mutations both reduced the response of the CRE reporter, whereas these substitutions either did not affect (L572^P6'^A) or modestly increased (M573^P7'^A) the response of the NFAT-RE reporter, arguing that Gα_s_ activation, but not Gα_q_ activation, selectively requires bulky hydrophobic residues at positions P6' and P7' for TA-dependent signalling in ADGRF1.

Strikingly, we found that the lack of signalling activity for F569^P3'^A in both NFAT-RE and CRE is accompanied by greatly decreased receptor abundance, as judged by the lack of a band on Western blot when probing for the N-terminal 3X-FLAG tag with an anti-Flag antibody (Fig. 5c). An antibody raised to a C-terminal portion (residues 831–880) of ADGRF1 showed a similar reduction in Western blot signal for the F569^P3'^A mutant (Supplementary Fig. 8), confirming that the loss of anti-FLAG reactivity was not due to proteolysis of the N-terminal tag. We propose that failure of the mutated TA to engage the 7TM leads to the exposure of the TA hydrophobic sequence, creating a neoepitope that stimulates heat-shock protein binding in the secretory pathway and subsequent degradation (i.e., the inactivity of the TA is the cause of the reduced protein amount, and not a consequence of it).

We explored the tolerance of F569^P3'^ more extensively by testing a suite of amino acid substitutions, including a conservative leucine substitution, a methionine substitution, and all of the charged amino acids (Fig. 5d, e). Among these substitutions, only the F569^P3'^L mutation produced a detectable signal (Fig. 5d), and only this substitution resulted in similar amounts of expressed protein as the wild-type CTF as judged by Western blot (Fig. 5e). The ability of F569^P3'^L to support TA-dependent signalling indicates that hydrophobic packing of this residue with F641^2.61^ is sufficient for signalling activity, and that an aromatic π-stacking interaction is not required.

Because of the variation in protein abundance for most of the F569^P3'^ mutants, we also assessed the role of the P3', P6' and P7' hydrophobic buried residues in activation using *trans* addition of TA peptide sequences, analogous to previous studies^10^. For these assays, we used a form of the 7TM domain lacking the TA, which we refer to as stalkless, or Δstalk. We incubated cells expressing the Δstalk form of ADGRF1 with a 12mer TA mimicking peptide (P12) at 100 µM or 10 µM, and with analogous peptides containing alanine substitutions at P3', P6' or P7', and measured signalling activity using the NFAT-RE reporter. We found that the Δstalk construct signalled robustly through NFAT-RE with the P12 peptide at 100 µM, but not 10 µM (Fig. 5f, g). Together, our data show that a large bulky hydrophobic residue is required at P3' for ADGRF1 tethered agonism, that TA-dependent Gα_q_ signalling is more tolerant of substitutions at the positions at P6' or P7' of the TA than Gα_s_ signalling, and that signal activation by peptides added in *trans* is intolerant of alanine substitutions at any of the TA positions (P3', P6', or P7') even for Gα_q_ signalling.

**Fig. 6:**
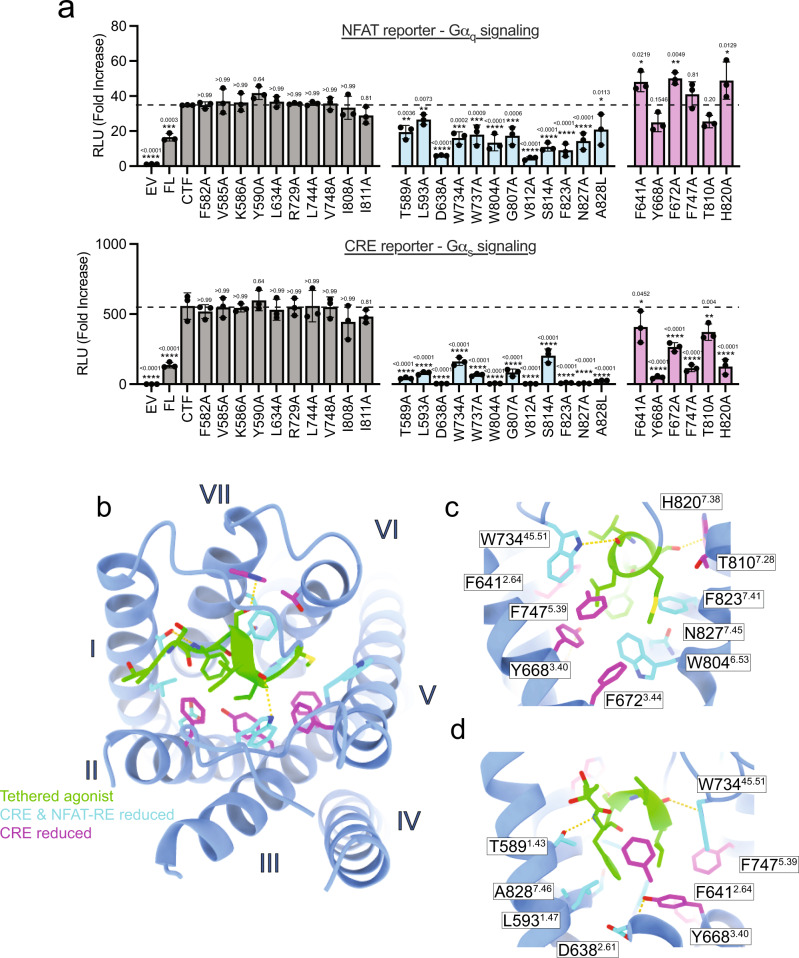
Binding site signalling determinants through NFAT-RE and CRE. **a** NFAT-RE and CRE signalling data for TA binding site mutants in the context of the ADGRF1 CTF protein. Cells were transfected with 20 ng of receptor DNA/well. Data are normalized to empty vector and reported as relative luminescence units (RLU). Data are grouped according to their effects on NFAT-RE and CRE signals relative to the wild-type ADGRF1 CTF protein. Mutations without significant effects on either reporter are grey, mutations that significantly reduce both NFAT-RE and CRE signals are cyan, and mutations that significantly reduce only the CRE signal are magenta. Data are presented as mean ± s.d. of three biological replicates. The mean for the CTF is indicated as a horizontal dotted line. Each point represents the mean value of three technical replicates. One-way analysis of variance (ANOVA) was used with Dunnett’s multiple-comparison post-hoc test to compare the difference between CTF to all other samples individually (*P < 0.05, **P < 0.01, ***P < 0.001 and ****P < 0.0001). **b** Top view of the TA in the orthosteric site. TA sidechain atoms and main-chain atoms making polar contacts (yellow dashes) are shown as sticks. Sidechains of 7TM residues that affect signalling when mutated to alanine are shown as sticks and are coloured according to the grouping in (**a**). **c** View from the P7' end and (**d**) the P3' end of the TA. Sidechains of 7TM residues that affect signalling when mutated to alanine are shown as sticks, coloured according to the grouping in (**a**), and annotated by residue with Wooten superscripts.

To further investigate TA-dependent activation of Gα_s_ and Gα_q_ by ADGRF1, we performed extensive alanine mutagenesis of the TA binding site in the 7TM domain and measured the signalling response of the NFAT-RE and CRE reporters. The signalling activity of the mutated proteins clustered into three distinct categories: mutations that did not affect the CRE or NFAT-RE response, those that were detrimental to both CRE and NFAT-RE, and those that were detrimental to CRE only (Fig. 6a, see Supplementary Fig. 9 for analysis of protein abundance by Western blot).

Residues where alanine substitutions reduced signalling in both the NFAT-RE and CRE reporters include conserved residues W734^45.51^ and W737 of ECL2, and several residues that surround F569^P3'^ in the 7TM core, including conserved ‘switch’ residue W804^6.53^, neighbouring residues F823^7.41^ and N827^7.45^, and T589A^1.43^, L593A^1.47^, A828L^7.46^ and D638A^2.61^ (Fig. 6b–d). These sets of mutations outline the importance of ECL2, the binding site arrangement around the ‘switch’ residue, and the conserved F569^P3'^ position for general ADGRF1 tethered agonism. We also found that positions V812^6.61^ and S814^6.63^ lowered signalling in both NFAT-RE and CRE assays, but this was likely due to a profound decrease in protein expression (Supplementary Fig. 9).

Strikingly, mutations that decreased CRE signalling but not NFAT-RE signalling were primarily in two separate clusters that interact with positions other than F569^P3'^ of the TA sequence. The first cluster contains residues within van der Waals contact of L572^P6'^ and M573^P7'^ at positions Y668^3.40^, F672^3.44^ and F747^5.39^ on TM helices III and V, with the F672^3.44^A substitution enhancing the NFAT-RE response (Fig. 6b–d). The second cluster consists of T810^7.28^ and H820^7.38^; T810^7.28^ interacts with M573^P7'^ and S574^P8'^, and H820^7.38^ approaches within H-bonding distance of S570^P4^. An additional site at which alanine substitution enhanced NFAT-RE signalling while reducing CRE signalling was F641^2.64^ on TM helix II. These results show that CRE signalling is more sensitive that NFAT-RE signalling upon disruption of the pocket proximal to P6' and P7', as well as upon disruption of interactions with S570^P4'^ and S574^P8'^.

## Discussion

For a typical GPCR in which the agonist is a soluble ligand, G protein coupling in a biological system is guided by intrinsic G protein binding preferences, agonist selectivity for G protein, and the local reservoir of G proteins in that cellular environment. aGPCRs are unique in that the 7TM bundle and its TA are encoded in the same polypeptide chain. As a result, aGPCR G protein signalling is simplified to a combination of two factors—the G protein preference of the TA-engaged CTF and the G protein context of the cellular environment.

**Fig. 7:**
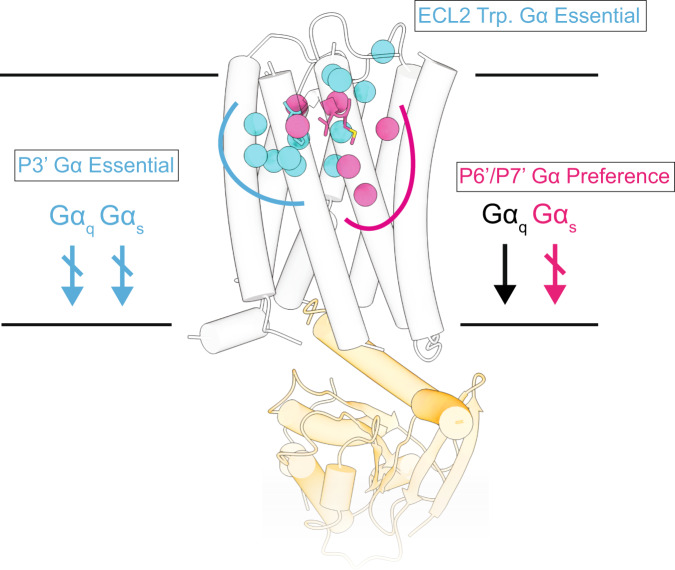
Mutations of the TA and associated binding site cluster by G protein class preference. ADGRF1 coupled to miniGα_s/q_ represented as tubed helices, coloured light grey and orange respectively. Residues that reduced signalling activity are shown, with TA residues rendered as sticks and with Cα atoms of residues in the TA binding site rendered as spheres. Residues that reduce both Gα_s_ and Gα_q_ signalling are coloured cyan, whereas residues that only reduce Gα_s_ signalling are magenta.

Here, we combine cryo-EM structural studies with mutational analysis and cell-based signalling assays to elucidate structure-function relationships in ADGRF1 and deduce its G protein coupling profile preferences. Our work confirms that ADGRF1 prefers coupling to Gα_q_, as reported previously^10^, provides a structure-based rationale for this coupling preference, and, most strikingly, identifies a cluster of residues in the TA and in the binding pocket that selectively suppress Gα_s_ coupling upon mutation while retaining Gα_q_ coupling (Fig. 7).

While the structure of the ADGRF1-miniGα_s/q_ complex shows that the overall architecture of the complex and the pose of the TA in the orthosteric site are similar to recently observed structures of ADGRF1 when complexed to miniGα_s_, miniGα_12/13_ and miniGα_i_^15^, there are subtle differences in the complexes that explain why Gα_q_ coupling is more robust than Gα_s_ coupling. Through extensive analysis of ADGRF1 signalling, we find that it is competent to signal through each G protein class at both the G protein proximal, and deep transcription factor readout level. This is an important confirmation, as the TRUPATH assays determine that all G protein classes undergo ADGRF1 dependent heterotrimer disassociation, and the transcription factor reporter assays show that ADGRF1 dependent signalling is of sufficient strength to propagate at the transcriptional activation level. We also establish that ADGRF1 can signal through Gα_12_ or Gα_13_, which to our knowledge makes ADGRF1 the first class B GPCR to show the capacity to signal through all major G protein classes^26^. Of note, these results are based on assays with overexpressed proteins. In future studies, it will be important to assess whether this capacity for ADGRF1 to couple to each G protein class leads to promiscuous signalling in specific physiological contexts.

Among the structural differences, tighter packing of L593^1.47^ with the highly conserved phenylalanine (P3') of the TA appears to be critical for Gα_q_ preference, because the positional shift of L593^1.47^ propagates its effects down helix I, introducing a hydrogen bond between S596^1.50^ and A828^7.48^ to stabilize the kink in TM helix VII. Our functional data suggests that these interactions contribute towards an increased stability of the ADGRF1-Gα_q_ complex relative to the Gα_s_ and Gα_i_ complexes. Both the TA and the binding pocket of the 7TM domain show increased mutational lability for Gα_s_ signalling at several residues, whereas Gα_q_ signalling remains unaffected by those same mutations, showing that Gα_q_ signalling is more tolerant to mutations than Gα_s_. Our data also confirm the critical importance of the F569^P3'^ residue for ADGRF1 G protein signalling, because a bulky hydrophobic residue at the P3' position is essential for expression and TA-dependent activation of the ADGRF1-CTF and mutations of residues that surround the F569^P3'^ pocket are detrimental in both NFAT-RE and CRE assays.

Unlike TA signalling of ADGRF1, which is tolerant of substitutions at positions other than F569^P3'^, we found that *trans* addition of 12mer peptides to a stalkless form of ADGRF1 mimics the TA only when the wild-type sequence is used at the relatively high concentration of 100 µM. Mutations at the P6' and P7' positions of the peptides, which are tolerated in TA signalling assays, failed to activate the stalkless ADGRF1 protein. This observation indicates that the development of peptidomimetic agonists for ADGRF1 will require extensive optimization if high potency is to be achieved in a soluble ligand.

Finally, our data elucidating a molecular basis for the preference of ADGRF1 for coupling to Gα_q_ over Gα_s_ identifies key sites in the 7TM that influence G protein coupling preferences. These findings should also inform the study of G protein coupling preferences and signalling biology for other aGPCRs.

## Methods

### MBP-CTF and signalling assay plasmids

DNA sequences encoding full length (20–910) and CTF (567–910) forms of ADGRF1 (Uniprot Q5T601) were subcloned into the pFuse-hIgG1-Fc2 vector behind an N-terminal IL2-signal sequence followed by a HA epitope tag, maltose binding protein and TEV protease site^23^. A FLAG tag was also inserted at the C-terminal end of the CTF before the stop codon. The CTF sequence was also subcloned to create the M-CTF protein with the N-terminal tags and mannose-binding protein omitted. Firefly reporter plasmids (CRE, NFAT-RE, SRE and SRF-RE) were assembled as described^23^. The pRL-TK plasmid (Promega) was used to express *Renilla* luciferase.

### Receptor and G protein plasmids

For recruitment assays, sequences encoding full-length (20–910) and CTF (567–910) ADGRF1 were subcloned into pcDNA3.1(+) with an N-terminal haemagglutinin signal sequence followed by a 3XFLAG purification tag and a TEV protease cleavage site. For the CTF construct the TEV protease site P1' residue encoded a threonine so that the correct residue would occupy T567 in the CTF (e.g., ENLYFQ/TSFSILM…). The C-terminus has a “GGGGSGGGGSSG” linker followed by a SmBiT tag “VTGYRLFEEIL”^31^. For proteins probed by Western blot the C-terminal SmBiT tag was replaced with a HA epitope tag. Mutagenesis was performed with a Q5 site directed mutagenesis kit (New England Biolabs) or using a modified QuikChange^TM^ method^32^.

MiniG protein sequences (miniGα_s_, miniGα_o_, miniGα_s/q_ and miniGα_12_) with capacity to complex with Gβ_1_γ_2_ (e.g. 399 variants^27^) were subcloned into pcDNA3.1(+) with an N-terminal LgBiT fusion followed by a “GGGGSGGGGSSGEF” linker that includes a translated EcoRI site. For structure determination and mutagenesis studies of ADGRF1, the constructs described above for recruitment assays were used.

### Protein expression, purification and complexation

ADGRF1 and miniGα_s/q_ were co-expressed in Expi293F cells. Cells were seeded at a density of 3.3 × 10^6^ cells/mL in 900 mL of Expi293 expression media. To transfect cells, equal amounts of receptor (250 µg) and miniG (250 µg) DNA were resuspended in 50 mL opti-MEM, FectoPro (Polyplus) transfection reagent (0.5 mL) was resuspended in 50 mL opti-MEM, the DNA and FectoPro solutions were mixed to give a final 1:1 DNA/FectoPro ratio, and the mixture incubated at room temperature for 10 min before adding to cells. Approximately 24 h later, filter-sterilised Valproic acid sodium salt (Sigma-Aldrich) was added to 5 mM, along with 10 mL of 45 % D-(+)-Glucose solution (Sigma-Aldrich). Cells were cultured for a further 24 h before they were harvested by centrifugation at 4000 *g* for 15 min. The pellet was flash frozen and then stored at −80 °C until purification.

Cells were thawed and resuspended in 250 mL of ice-cold hypotonic lysis buffer (10 mM HEPES, pH 7.5, containing Roche protease inhibitor tablets and 0.1 mU/mL apyrase to prevent separation of the GPCR-miniG complex). The pellet was stirred on ice for 30 min and lysed by dounce homogenization. The broken cells were pelleted by centrifugation at 50,000 *g* and resuspended in 225 mL solubilisation buffer (10 mM HEPES, pH 7.5 containing 1 mM MgCl_2_, 2 mM CaCl_2,_ and 100 mM NaCl) with 0.1 mU/mL apyrase and 1:100,000 Benzonase (v/v). The resuspended material was dounce homogenized, and DDM-CHS (10:1 pre-mix, Anatrace) was then added to a final concentration of 1% (w/v). This solution was stirred for 75 min at 4 °C, clarified by centrifugation at 50,000 *g* for 1 h, and passed through a glass microfibre filter. The filtrate was then loaded onto M2 anti-FLAG antibody affinity resin by gravity flow. The resin was washed with 30 column volumes of wash buffer (10 mM HEPES, pH 7.5 containing 1 mM MgCl_2_, 2 mM CaCl_2,_ 100 mM NaCl, and 0.1 % (w/v) LMNG-CHS), and the protein was eluted by the application of wash buffer containing 100 µg/mL of 3XFLAG peptide. The eluted protein was concentrated using a 100 kDa MWCO centrifugal concentrator (Amicon).

Nb35 and human Gβ_1_γ_2_ were purified according to previously published protocols^33^. ADGRF1-miniGα_s/q_, Nb35 and Gβ_1_γ_2_ were mixed in a 1:1.15:1.15 ratio and incubated overnight at 4 °C with rotation. The protein mixture was centrifuged at 21,000 *g* for 20 min at 4 °C, and then spun through a Durapore® PVDF 0.1 µm column (Millipore). The protein mixture was injected onto a Superdex S200 10/300 GL column equilibrated in SEC buffer (10 mM HEPES pH 7.5 containing 1 mM MgCl_2_, 2 mM CaCl_2,_ 100 mM NaCl, and 0.005 % (w/v) LMNG-CHS). The peak fraction containing the complex was collected and concentrated using a 100 kDa MWCO centrifugal concentrator to ~8 mg/mL.

### Cryo-EM sample preparation and image acquisition

The complex was vitrified on QUANTIFOIL® holey carbon grids (400-mesh, copper, R1.2/1.3, Electron Microscopy Sciences) using a FEI Vitrobot Mark IV (FEI, Hillsboro). Grids were glow-discharged, and 3 µL of sample loaded to the grid in a chamber at 22 °C and 100 % humidity. Samples were applied at a force of 15 and blotted for 5–7 s before plunge-freezing in liquid ethane.

Data were collected using a FEI Titan Krios at 300 kV with a Gatan Quantum Image Filter with K3 Summit direct electron detection camera in counting mode with a total exposure dose of ~54 e^−^/Å^−2^. Thermo Scientific Smart EPU software was used for acquisition. 50 frames per movie were collected at magnification of 105,000x, corresponding to 0.825 Å per pixel. Micrographs were collected at defocus values ranging from −0.5 to −1.5 µm. The processing scheme is summarised in Supplementary Fig. 4; in brief, motion corrected and dose-weighted using the RELION motion correction implementation^34^ and contrast transfer function parameters estimated by CTFFIND4^35^. Low quality micrographs were removed using micassess^36^ prior to particle picking in topaz^37^. ~4.9 M particles were initially identified, leading to ~2.9 M particles after 2D classification in RELION^38^. Two independent 3D classifications in RELION were then performed with different initial references, with the best classes from each subsequently combined and duplicates removed to leave ~1.2 M particles corresponding to ADGRF1. Following further 3D classification in RELION ~ 660,000  particles underwent particle polishing in RELION^34^ and further 2D classification followed by masked 3D classification with no alignment using a T value of 20 in RELION. 73,903 particles with strong transmembrane density were identified which resulted in a 3.6 Å reconstruction from CryoSPARC non-uniform refinement with contrast transfer function refinement^39^. Subsequent local non-uniform refinement with a mask excluding the micelle improved the reconstruction to 3.44 Å.

### Atomic modelling and model refinement

Model building was carried out in Coot using overlaid non-uniform refinement and local filtered maps generated in CryoSPARC^40^, as well as deepEMhancer^41^ maps. The ADGRF1-miniGα_s_ coordinate file (PDB: 7WU3) was used as an initial model. The miniGα_s_ sequence was changed in Coot to generate miniGα_s/q_^27^. The coordinates were then manually rebuilt using Coot, and refined using ISOLDE^42^, Phenix Real-Space Refine^43^ and Servalcat^44^. The final models were evaluated using MolProbity. Statistics of the map reconstruction and model refinement are presented in Table 1. Structural biology applications used in this project (except CryoSPARC) were compiled and configured by SBGrid^45^. Molecular graphics and analyses performed with UCSF ChimeraX, developed by the Resource for Biocomputing, Visualization, and Informatics at the University of California, San Francisco, with support from National Institutes of Health R01-GM129325 and the Office of Cyber Infrastructure and Computational Biology, National Institute of Allergy and Infectious Diseases^46,47^.

**Table 1:** Cryo-EM data collection, refinement, and validation statistics

**ADGRF1**	
EMD-29684	
PDB 8G2Y	
**Data collection and processing**	
Voltage (kV)	300
Electron exposure (e^−^/Å^−2^)	~53.5
Defocus range (μm)	0.5–1.5
Pixel size (Å)	0.825
Symmetry imposed	C1
Initial particle images (no.)	4,960,830
Final particle images (no.)	73,903
Map Resolution (Å)	3.44
FSC threshold	0.143
Map resolution range (Å)	3.1–6.0
**Refinement**	
Initial model used	7wu3
Model resolution (Å)	3.10
FSC threshold	0.143
Map-sharpening B-factor (Å^2^)	−111.2
Model composition	
Non-hydrogen atoms	6,676
Protein residues	891
B factors (Å^2^)	
Protein	74.10
Ligand	98.71
R.M.S.D. deviations	
Bond lengths (Å)	0.005
Bond angles (°)	1.125
**Validation**	
MolProbity score	1.55
Clashscore	4.67
Poor rotamers (%)	0.00
Ramachandran plot	
Favoured (%)	95.47
Allowed (%)	4.53
Disallowed (%)	0.00

### Cell culture

HEK293T (ATCC, CRL-3216) and HEKΔ6 (from Asuka Inoue, Tohoku University) cells were cultured in DMEM supplemented with 10% FBS and 1% penicillin-streptomycin at 37 °C in a 5% CO_2_ humidified incubator.

### Western blot analysis

To assess target protein expression and a loading control concurrently, we used two imaging channels on a LI-COR Odyssey CLx Fluorescent Imaging System. Antibodies were validated by both external sources and with comparison to empty vector controls in all western blots. To probe protein expression via the 3XFLAG epitope, we incubated membranes with 1:10,000-fold dilution of anti-FLAG M2 mouse antibody (Sigma-Aldrich F3165) and a 1:10,000 anti-GAPDH rabbit antibody (Cell Signalling Technology 14C10) as loading control. To probe protein expression via a C-terminal region of ADGRF1 (831–880) we incubated the membrane with 1:1,000-fold dilution of an anti-ADGRF1 rabbit antibody (Sigma-Aldrich SAB4501161) and 1:1,000-fold dilution of an anti-β-tubulin mouse antibody (Cell Signalling technology D3U1W) as a loading control. To visualise 1:10,000-fold dilutions of Donkey anti-rabbit IRDye®680 and goat anti-mouse IRDye®800 fluorophore conjugated secondary antibodies (LI-COR) were incubated with membranes.

### Dual luciferase reporter assays

HEK293T cells were seeded in DMEM in clear-bottom white 96-well plates pre-coated with poly-D-lysine the day before transfection. At roughly 70 % cell confluency, the medium in each well was replaced with fresh DMEM. Cells were transfected using Lipofectamine^TM^ 2000 (Invitrogen) at a ratio of 3 µL per 1 µg DNA in Opti-MEM using the manufacturer’s instructions. Each well was co-transfected with 50 ng firefly reporter plasmid for CRE and NFAT-RE along with 1 ng pRL-TK plasmid. For SRE and SRF-RE each well was co-transfected with 30 ng of firefly reporter plasmid and 0.6 ng pRL-TK plasmid. Cells were also co-transfected with the indicated amounts of receptor DNA. Cells were cultured in the transfection mixture for 6 h before the media was replaced with complete media. 24 h after transfection, the media was replaced with fresh DMEM, and the cells were incubated for another 8 h prior to assay readout. For SRF-RE and SRE read-outs the media was replaced with DMEM lacking FBS. Before measuring luminescence, cells were washed once with PBS, 20 µL of 1X Passive Lysis Buffer was added to each well, and the lysates were incubated with shaking for 15 min. Cell lysates were serially pipetted, and the assay plate was centrifuged at 400 *g* for 1 min. Dual-Glo luciferase measurements (Promega) were read on a GloMax luminometer. The ratio of Firefly: Renilla signal was calculated for each well and normalized to the mean of the EV-transfected controls to give Relative Luminescence Units (RLU). Each assay was performed in technical triplicate wells and then repeated in biological triplicate on different days.

For peptide experiments, transfection was performed as above. Peptides were dissolved in DMSO to give 10 mM stocks and diluted to 10x concentration in Opti-MEM immediately before use. The final DMSO concentration was 1 % (v/v) in all wells. Peptides were added in trans at the initial media change 6 h after transfection, and luminescence readings were acquired 24 h after transfection. Each assay was performed in technical triplicate wells and then repeated in biological duplicate on different days.

For Gα rescue experiments in HEKΔ6 cells, the standard CRE, NFAT-RE, and SRF-RE assay formats described for wild-type cells were used, with the exception that DNA encoding Gα subunits was also included in the transfection procedure. Per well, Gα DNA loads are as follows. For the CRE assay: 1 ng of Gα_s_; 20 ng of Gα_q_, Gα_11_, Gα_16_, Gα_12_, and Gα_13_. For the NFAT-RE assay: 20 ng of Gα_s_, Gα_q_, Gα_11_, and Gα_16_; 1 ng of Gα_12_, and Gα_13_. For the SRF-RE assay: 20 ng of Gα_s_, Gα_q_, Gα_11_, and Gα_16_; 0.3 ng of Gα_12_, and Gα_13_. Total DNA per well was balanced with pcDNA3.1. Each assay was performed in technical triplicate wells and then repeated in biological duplicate on different days.

### G protein NanoBiT recruitment assays

HEK293T cells were seeded in DMEM in clear-bottom white 96-well plates pre-coated with poly-D-lysine the day before transfection. Cells were transfected using GeneJuice^TM^ (Sigma-Aldrich) at a ratio of 3 µL per 1 µg DNA in Opti-MEM using manufacturer’s instructions. Equal amounts of receptor and miniG DNA (50 ng) were used per well. Cells were incubated for 48 h and washed with PBS prior to assay readout. Cells were resuspended in opti-MEM and the Nano-Glo® Luciferase Assay was read out on a GloMax luminometer. Each assay was performed in biological triplicate.

### TRUPATH G protein signalling assays

TRUPATH plasmids were a generous gift from Dr. Justin English, University of Utah. TRUPATH plasmids encode the same protein sequences reported in the original paper^25^, but genes encoding Gα-*Renilla*, Gβ_1_ and GFP-γ_2_ are encoded on a single plasmid. Sequences encoding the Gα-*Renilla* and Gβ_1_ genes have a triple T2A sequence between them; the Gβ_1_ gene and GFP-γ_2_ are separated by an intervening IRES sequence.

For the TRUPATH assays, HEK293T cells were seeded in DMEM in clear-bottom white 96-well plates pre-coated with poly-D-lysine the day before transfection. Cells were transfected with 80 ng of TRUPATH plasmid and 20 ng of receptor plasmid per well. The culture media was changed 4–6 h after transfection, and BRET measurements were taken 48 h after transfection. To record the BRET signal, the media was removed from the wells, and cells were then washed twice with Hank’s Balanced Salt Solution (HBSS) containing 10 mM HEPES, pH 7.5. Coelenterazine 400a (NanoLight Technology) was reconstituted to 1 mM in anhydrous ethanol and frozen at −80 °C. Immediately prior to BRET measurement, coelenterazine 400a was diluted to 6 µM working solution in HBSS and 80 µL was applied to the cells. BRET was measured using a GloMax luminometer using ET405/40x and ET510lp filters (Chroma Technology Corp), for donor (*Renilla)* and acceptor (GFP) readouts respectively.

### Reporting summary

Further information on research design is available in the Nature Portfolio Reporting Summary linked to this article.

## Supplementary information

Supplementary Information

Reporting Summary

## Data Availability

Source data are provided with this paper. The Cryo-EM data, including unprocessed and processed maps, generated in this study have been deposited in the Electron Microscopy Databank under the accession code EMD-29684. The modelled protein structure generated in this study has been deposited at the Protein Data Bank under the accession code 8G2Y. All other data are available from the corresponding author on request. Source data are provided with this paper.

## Source data

Source Data
