# Supplementary material for: Tethered agonist activated ADGRF1 structure and signalling analysis reveal basis for G protein coupling
Source: Nat Commun. 2023 Apr 29;14:2490. doi: 10.1038/s41467-023-38083-7 (PMC10148833; doi:10.1038/s41467-023-38083-7)
Supplement: Supplementary file 1 — Supplementary Information [file 41467_2023_38083_MOESM1_ESM.pdf]

Supplementary information

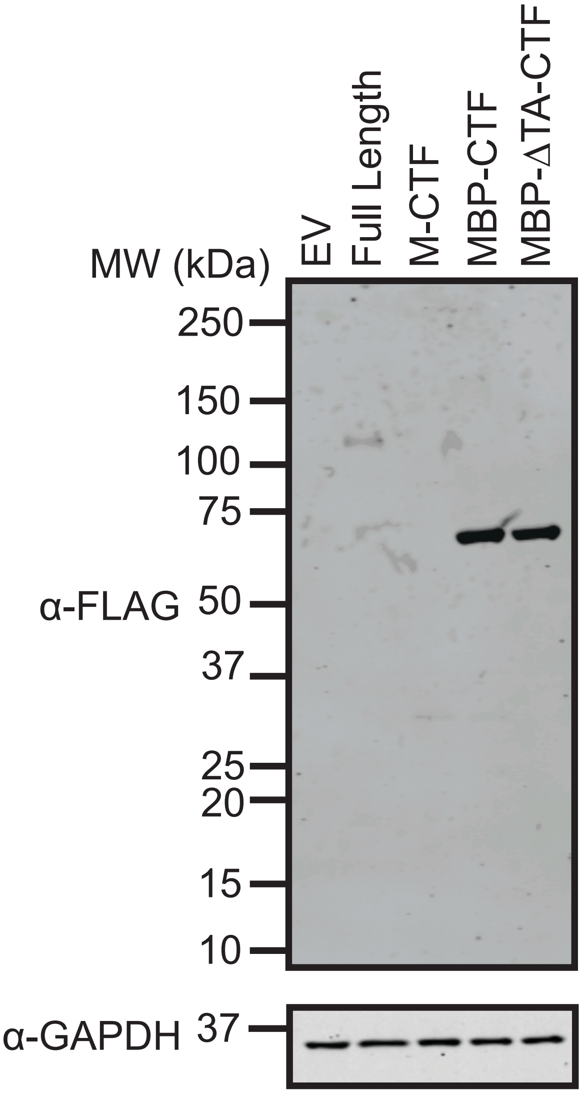


**Supplementary Fig. 1: Expression of ADGRF1 proteins used in signalling experiments with transcription factor reporters and TRUPATH biosensors.** α-FLAG Western blot of ADGRF1 proteins used in signalling assays to identify G protein coupling partners in transcription factor reporter and TRUPATH assays. A Western blot with an anti-GAPDH antibody was used as a loading control. An example blot is shown as a representative of three biological repeats.

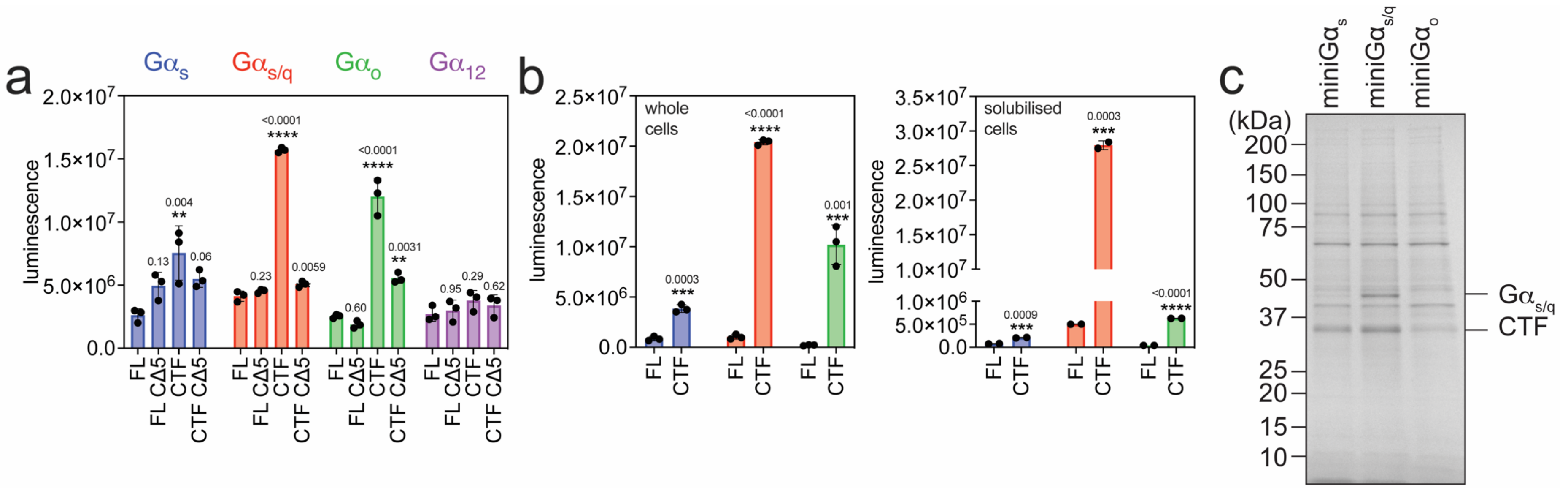


**Supplementary Fig. 2: NanoBiT complementation of ADGRF1-CTF with miniGα proteins.** (a) NanoBiT recruitment assays for assessment of receptor-miniG coupling efficiency. Adherent HEK293T cells were transfected with equal amounts of DNA for receptor and miniG proteins and luminescence recorded after the addition of substrate. Full-length (FL) and C-terminal fragment (CTF) variants of the receptor were combined with either functional miniG or non-functional miniG CΔ5 controls. Luminescence resulting from luciferase reconstitution is plotted, with bars grouped and coloured by miniG class. Data are presented as mean ± s.d. of three biological replicates. One-way analysis of variance (ANOVA) was used with Dunnett's multiple-comparison post-hoc test to compare the difference between FL to all other samples within each miniG group individually (**P*≤.05, ***P*≤.01, ****P*≤.001 and *****P*≤.0001). (b) Suspension Expi293T cells were transfected with equal amounts of DNA for receptor and miniG proteins in whole cell (left) and solubilised formats (right). Luminescence resulting from luciferase reconstitution is plotted, with bars coloured by miniG class as in (a). Data are presented as mean ± s.d. of three biological replicates for whole cell analysis, and two biological replicates for solubilised cell analysis. Unpaired two-tailed T-test was used to compare the difference between FL and CTF for whole cell and solubilised cell luminescence (****P*≤.001 and *****P*≤.0001). (c) Coomassie blue staining after SDS-PAGE of ADGRF1-CTF complexes recovered on magnetic beads, assessing coupling efficiency to miniG proteins.

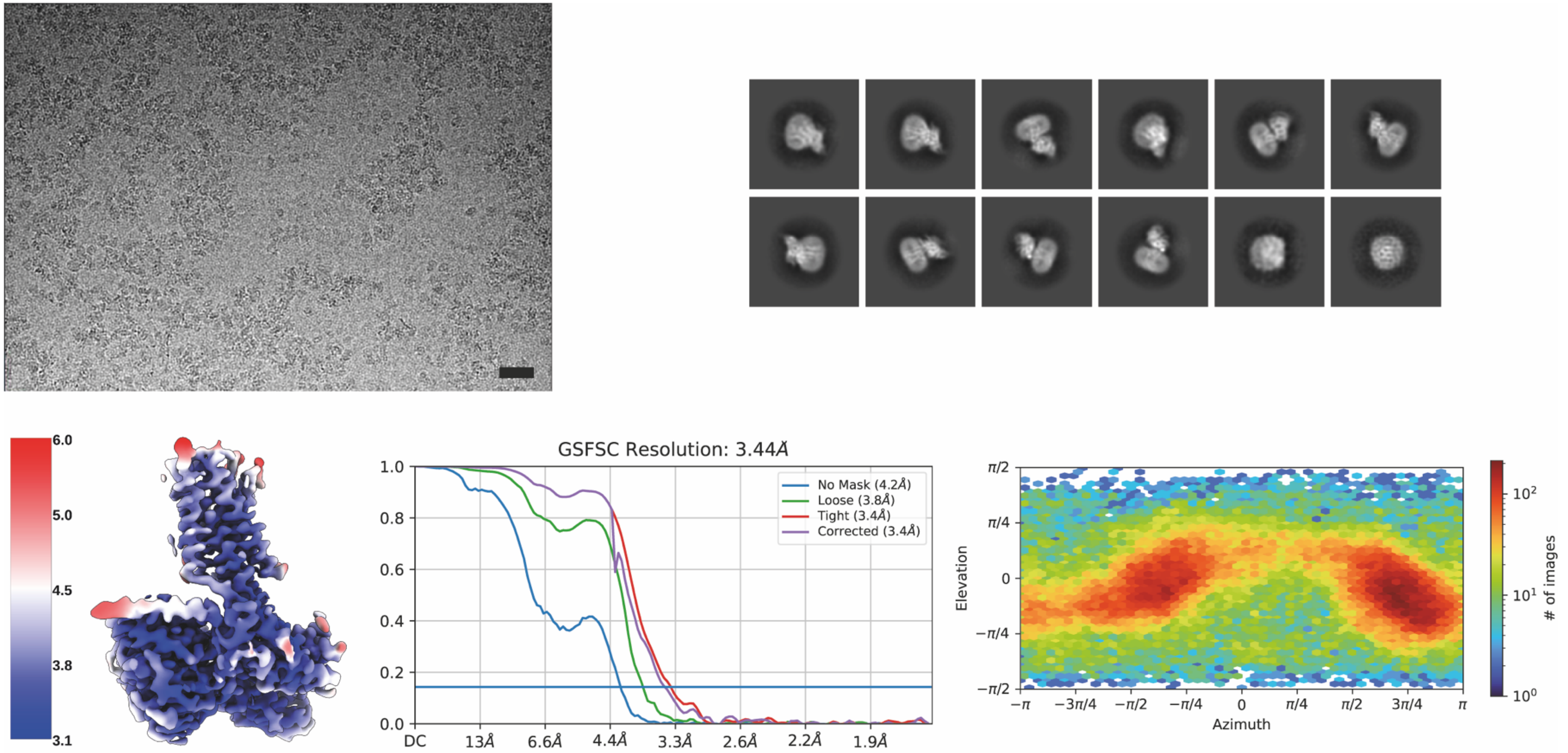


**Supplementary Fig. 3: Cryo-EM data quality.** (Top, left) A representative micrograph from a total of 7310 micrographs of ADGRF1 complexes in vitreous ice visualized by cryo-EM on a Titan Krios microscope equipped with Gatan K3 detector. Scale bar indicates 300 Å. (Top, right) 2D-class averages showing a range of particle orientations. (Bottom, left) Local resolution map generated in Cryosparc (GS-FSC = 0.143 cut-off). (Bottom, centre) GS-FSC curves with default Cryosparc masks. (Bottom, right) Particle orientation distribution map of ADGRF1-miniG$\alpha_{s/q}$ complexes used for structure determination.

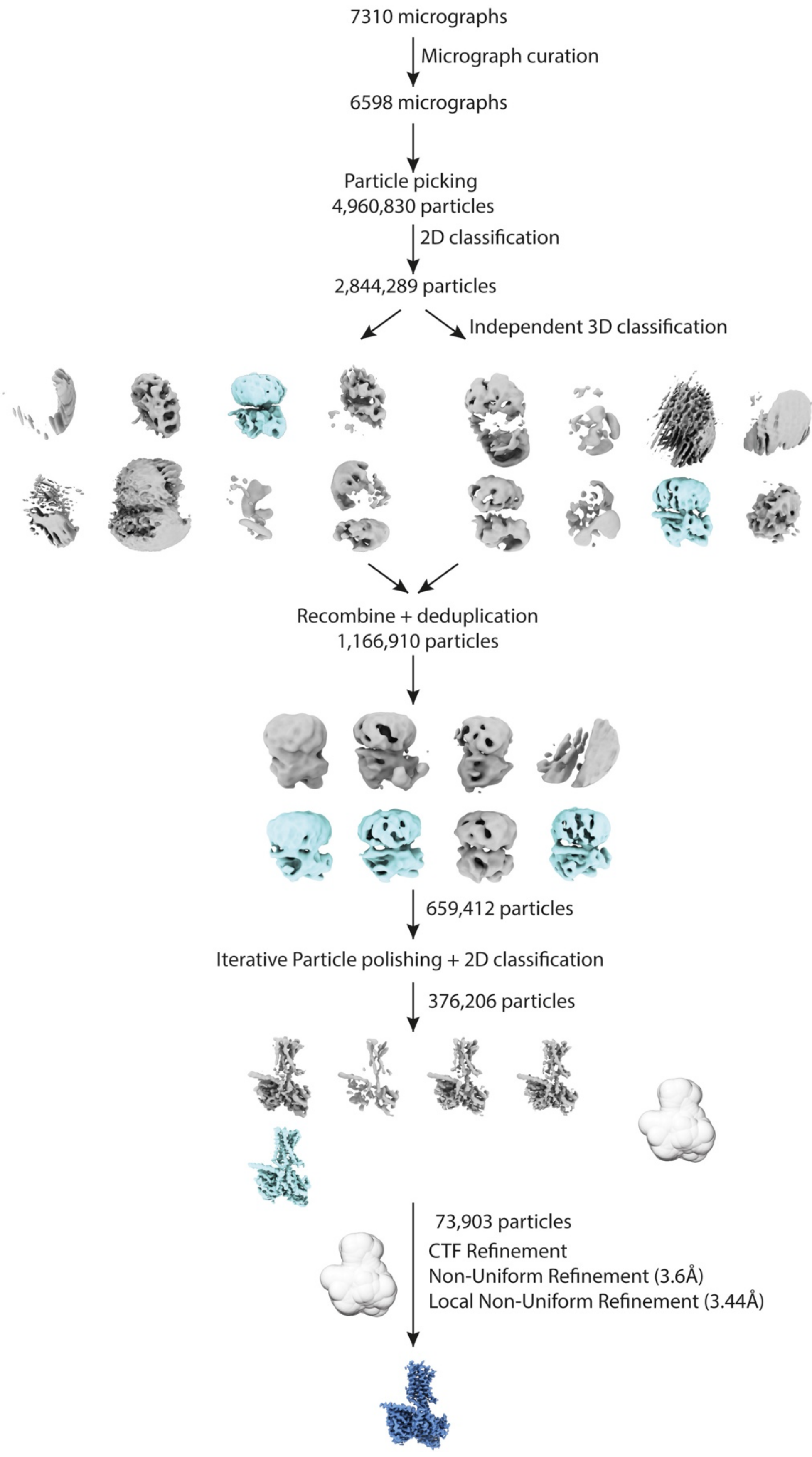


**Supplementary Fig. 4: Cryo-EM processing scheme for ADGRF1 reconstruction.** Selected classes are blue and discarded classes are grey.

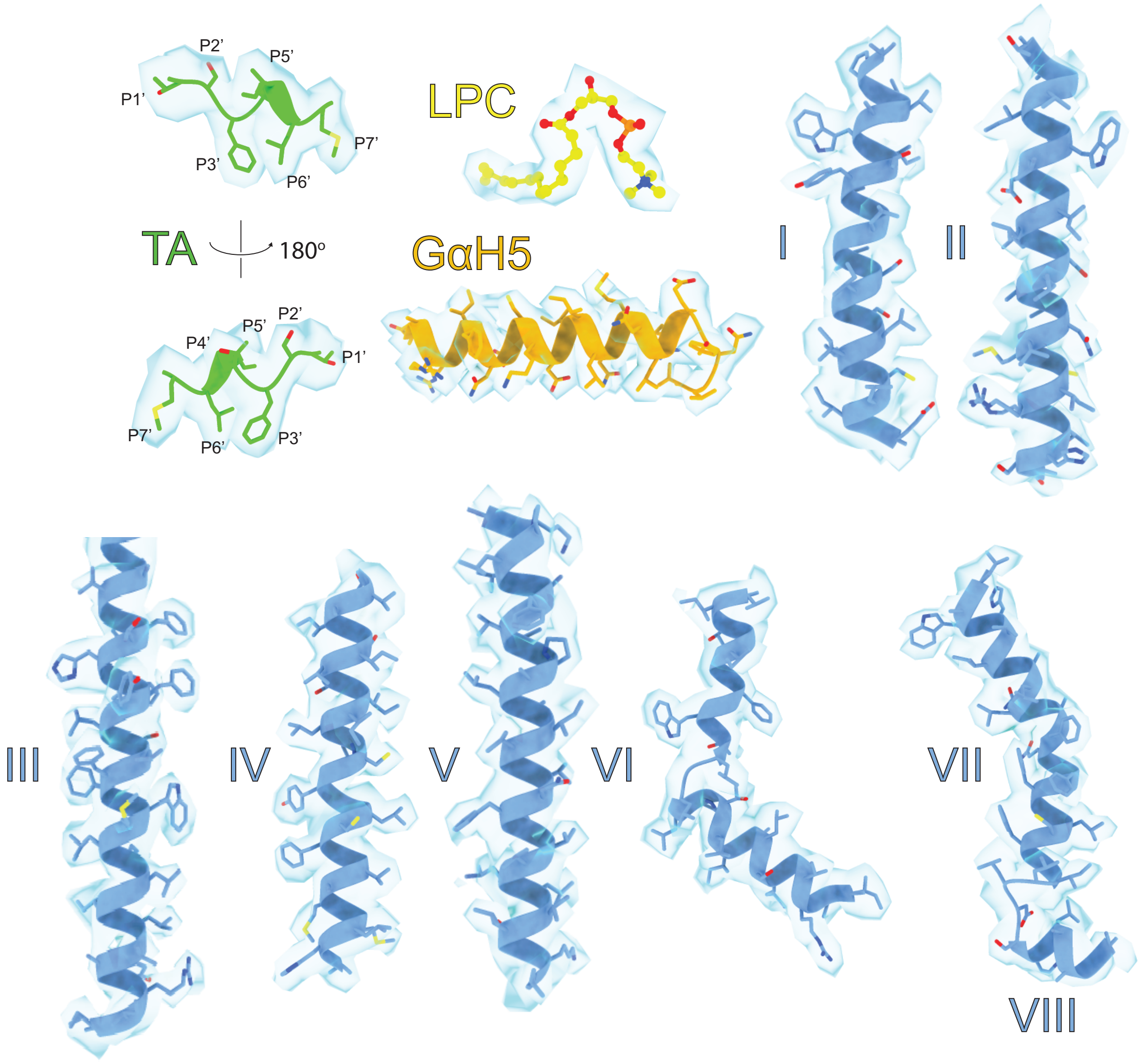


**Supplementary Fig. 5: Map to model density of key ADGRF1 components.** All protein structures are rendered in cartoon with residue side chains represented as sticks. The LPC molecule is rendered as ball-and-sticks. TA (green); tethered agonist; LPC (yellow), lysophosphatidylcholine (16:0), GαH5 (orange); C-terminal alpha helix of miniG$\alpha_{s/q}$, I-VII (blue) transmembrane helices I-VII, and VIII (blue); helix VIII. The density threshold was set at three standard deviations from the mean for all components.

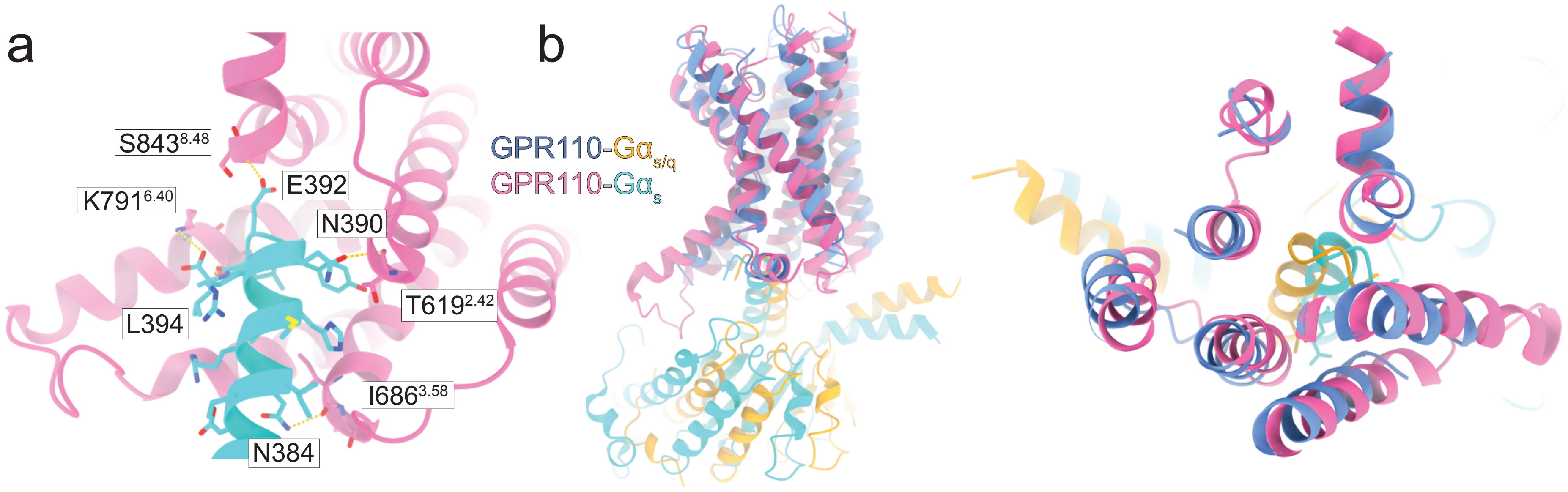


**Supplementary Fig. 6: Comparison between ADGRF1 bound to miniG$\alpha_{s/q}$ and miniG$\alpha_s$.** a) Close-up of the ADGRF1 (pink) interface with G$\alpha_s$ (cyan) along the C$\alpha$5 helix of G$\alpha_s$. Hydrogen bonds between the receptor and G$\alpha$ are indicated. (b) Left: side-view, and Right: top-slice view of ADGRF1 bound to miniG$\alpha_s$ or miniG$\alpha_{s/q}$. The receptors are aligned by least-squares superposition of TM helices I and VII.

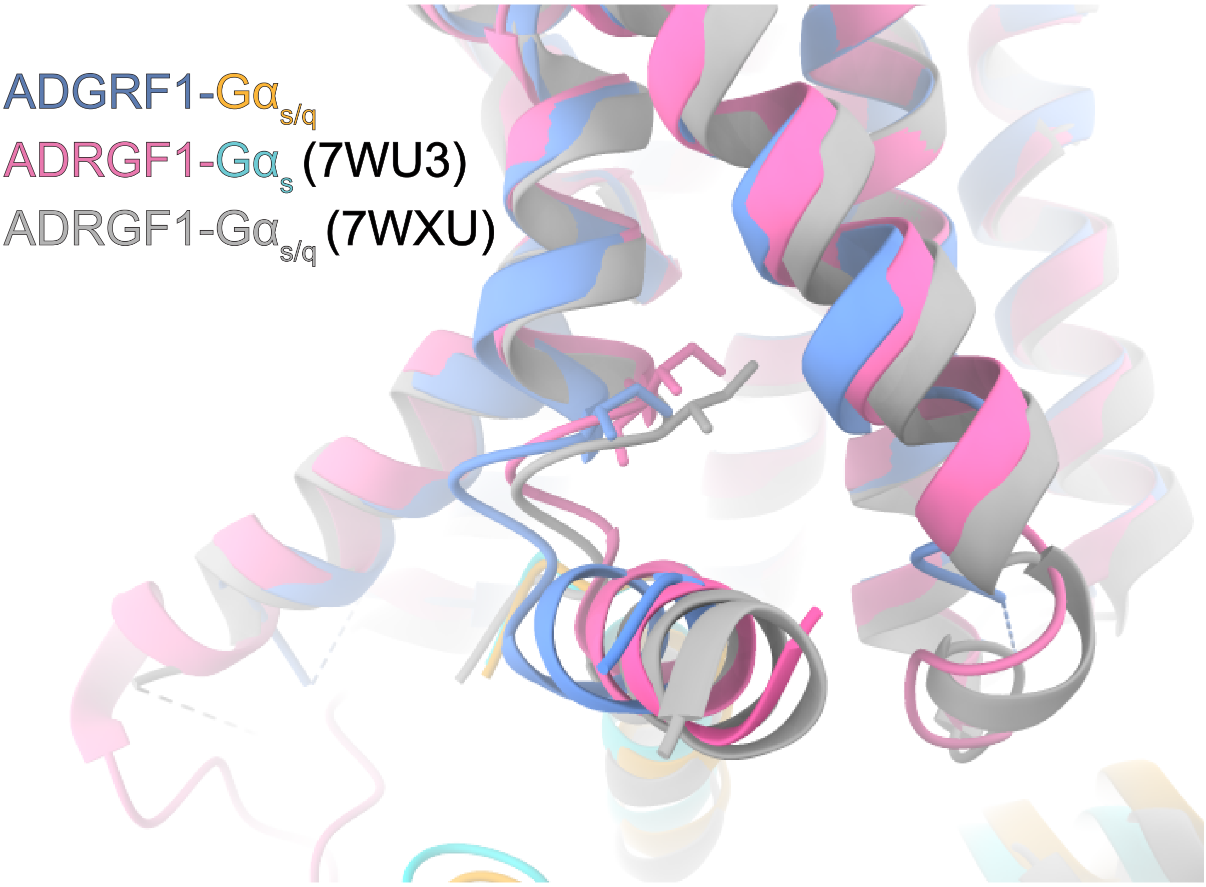


Supplementary Fig. 7: Comparisons of the structure of the complex between ADGRF1 bound to miniGα$_{s/q}$ determined in this work with the structure determined using HiBiT-LgBiT tethering (PDB ID code 7WXU), and the ADGRF1 miniGα$_s$ structure (PDB ID code 7WU3).

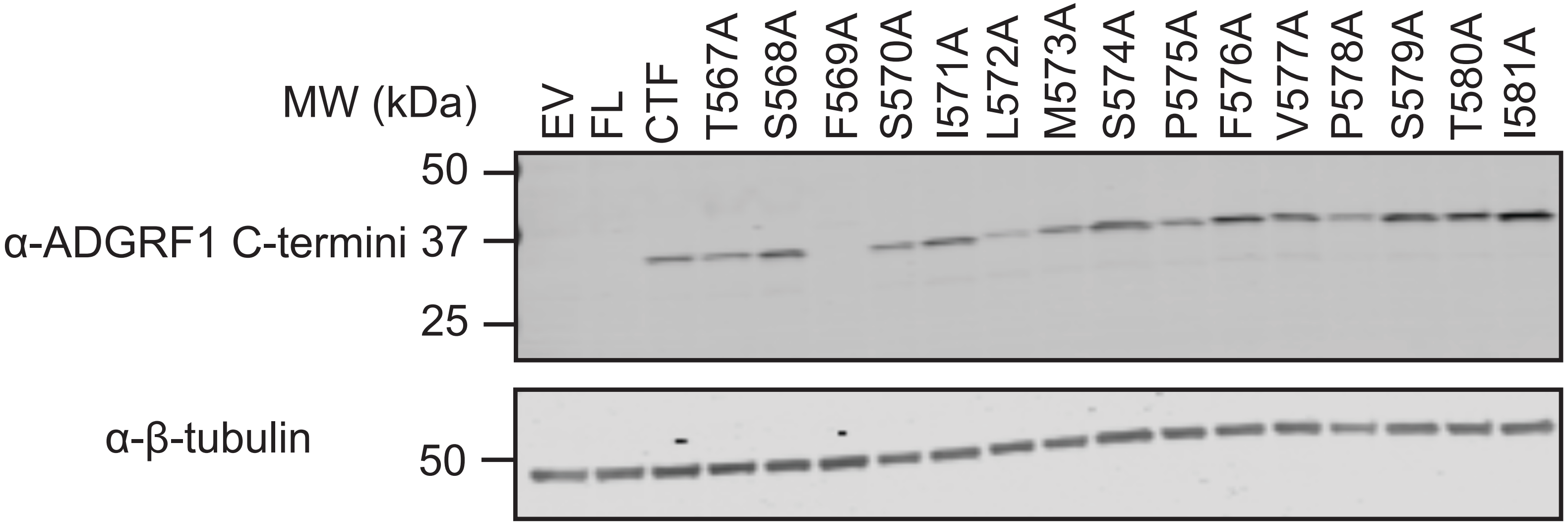


**Supplementary Fig. 8: Amount of ADGRF1 CTF protein expressed by wild-type and alanine scanning mutants.** Protein was detected by Western blotting with an α-ADGRF1 C-terminal antibody. An example blot is shown as a representative of two biological repeats.

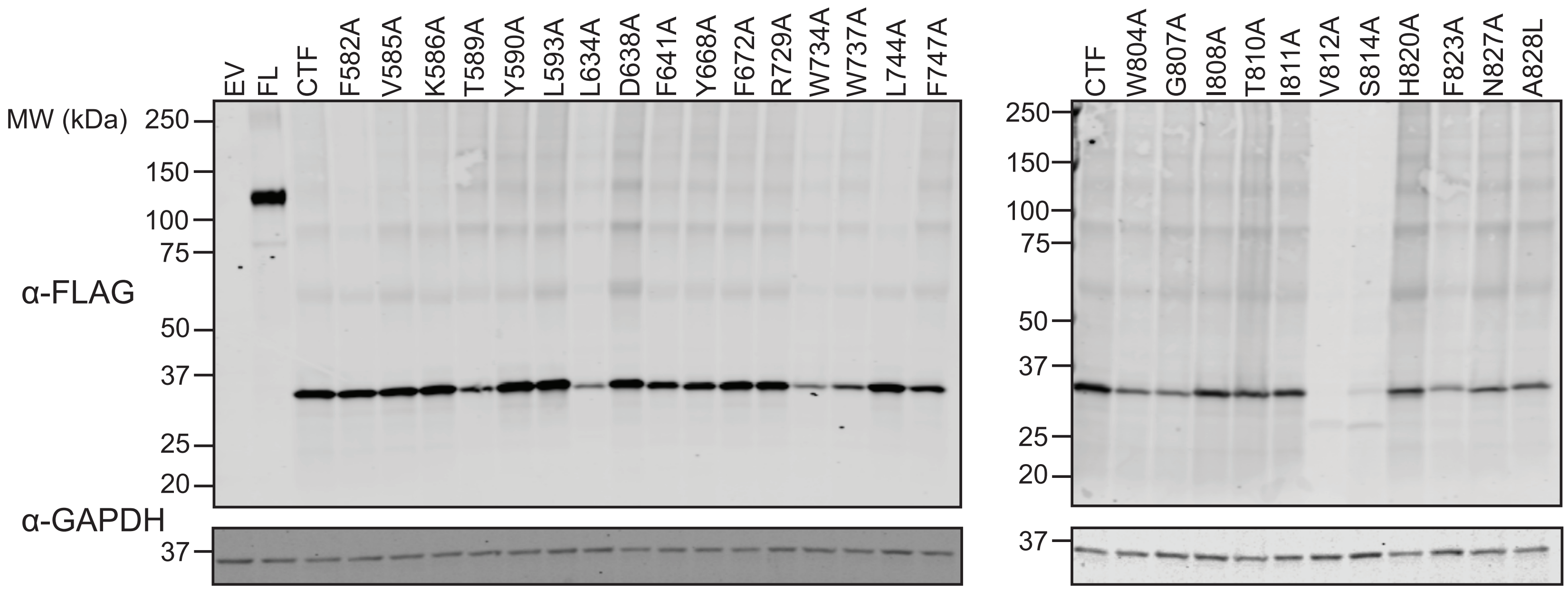


**Supplementary Fig. 9 – Amount of ADGRF1 CTF protein expressed by wild-type and alanine scanning mutants of the binding site.** Protein was detected by Western blotting with an α-FLAG antibody, which recognizes an N-terminal FLAG tag. A Western blot with an anti-GAPDH antibody was used as a loading control. A Western blot with an anti-GAPDH antibody was used as a loading control. An example blot is shown as a representative of two biological repeats.
