# Supplementary material for: Tethered agonist activated ADGRF1 structure and signalling analysis reveal basis for G protein coupling
Source: Nat Commun. 2023 Apr 29;14:2490. doi: 10.1038/s41467-023-38083-7 (PMC10148833; doi:10.1038/s41467-023-38083-7)
Supplement: Supplementary file 2 — Reporting Summary [file 41467_2023_38083_MOESM2_ESM.pdf]

nature portfolio

Corresponding author(s): Stephen C. Blacklow
Daniel T. D. Jones

Last updated by author(s): Wed 5th October 2022

Reporting Summary

Nature Portfolio wishes to improve the reproducibility of the work that we publish. This form provides structure for consistency and transparency in reporting. For further information on Nature Portfolio policies, see our Editorial Policies and the Editorial Policy Checklist.

Statistics

For all statistical analyses, confirm that the following items are present in the figure legend, table legend, main text, or Methods section.

n/a	Confirmed	
☐	☒	The exact sample size (*n*) for each experimental group/condition, given as a discrete number and unit of measurement
☐	☒	A statement on whether measurements were taken from distinct samples or whether the same sample was measured repeatedly
☐	☒	The statistical test(s) used AND whether they are one- or two-sided<br>*Only common tests should be described solely by name; describe more complex techniques in the Methods section.*
☐	☒	A description of all covariates tested
☐	☒	A description of any assumptions or corrections, such as tests of normality and adjustment for multiple comparisons
☐	☒	A full description of the statistical parameters including central tendency (e.g. means) or other basic estimates (e.g. regression coefficient) AND variation (e.g. standard deviation) or associated estimates of uncertainty (e.g. confidence intervals)
☐	☒	For null hypothesis testing, the test statistic (e.g. *F*, *t*, *r*) with confidence intervals, effect sizes, degrees of freedom and *P* value noted<br>*Give P values as exact values whenever suitable.*
☒	☐	For Bayesian analysis, information on the choice of priors and Markov chain Monte Carlo settings
☒	☐	For hierarchical and complex designs, identification of the appropriate level for tests and full reporting of outcomes
☒	☐	Estimates of effect sizes (e.g. Cohen's *d*, Pearson's *r*), indicating how they were calculated

Our web collection on statistics for biologists contains articles on many of the points above.

Software and code

Policy information about availability of computer code

Data collection	EPU v-2.2.0
Data analysis	Relion 3.1, CryoSPARC 2.15, Pymol 2.32, Coot 0.89, Phenix 1.18.2. MotionCor2, CTFFIND4.1, UCSF Chimera 1.15, MolProbity 4.1, UCSF ChimeraX 1.3, deepEMhancer, MicAssess, Servalcat 0.2.

For manuscripts utilizing custom algorithms or software that are central to the research but not yet described in published literature, software must be made available to editors and reviewers. We strongly encourage code deposition in a community repository (e.g. GitHub). See the Nature Portfolio guidelines for submitting code & software for further information.

Data

Policy information about availability of data

All manuscripts must include a data availability statement. This statement should provide the following information, where applicable:

- Accession codes, unique identifiers, or web links for publicly available datasets
- A description of any restrictions on data availability
- For clinical datasets or third party data, please ensure that the statement adheres to our policy

The Cryo-EM data, including unprocessed and processed maps, generated in this study have been deposited in the Electron Microscopy Databank under the accession code EMD-29684 [https://www.ebi.ac.uk/emdb/EMD-29684]. The modelled protein structure generated in this study has been deposited at the Protein Data Bank under the accession code 8G2Y [http://doi.org/10.2210/pdb8G2Y/pdb].

Human research participants

Policy information about studies involving human research participants and Sex and Gender in Research.

Reporting on sex and gender	N/A
Population characteristics	N/A
Recruitment	N/A
Ethics oversight	N/A

Note that full information on the approval of the study protocol must also be provided in the manuscript.

Field-specific reporting

Please select the one below that is the best fit for your research. If you are not sure, read the appropriate sections before making your selection.

☒ Life sciences ☐ Behavioural & social sciences ☐ Ecological, evolutionary & environmental sciences

For a reference copy of the document with all sections, see nature.com/documents/nr-reporting-summary-flat.pdf

Life sciences study design

All studies must disclose on these points even when the disclosure is negative.

Sample size	For all assays, at least two replicates, with three in most cases, were carried out and sufficient for the observations and claims made in this study. See figure legends for details.
Data exclusions	No data were excluded.
Replication	Functional experiments were carried out at least two times independently, with all attempts at replication successful.
Randomization	Randomization was not necessary. All independent variables and associated observations were sufficient for the interpretation and claims made within this study.
Blinding	Blinding was not necessary in this study. The cryo-EM map is derived from thousands of particles that were independently classified into discrete volumes, without any *a priori* designation. Blinding was also not necessary for experimental data acquired in this study, and all data is subject to statistical analysis when necessary.

Reporting for specific materials, systems and methods

We require information from authors about some types of materials, experimental systems and methods used in many studies. Here, indicate whether each material, system or method listed is relevant to your study. If you are not sure if a list item applies to your research, read the appropriate section before selecting a response.

Materials & experimental systems

n/a	Involved in the study	
☐	☒	Antibodies
☐	☒	Eukaryotic cell lines
☒	☐	Palaeontology and archaeology
☒	☐	Animals and other organisms
☒	☐	Clinical data
☒	☐	Dual use research of concern

Methods

n/a	Involved in the study	
☒	☐	ChIP-seq
☒	☐	Flow cytometry
☒	☐	MRI-based neuroimaging

Antibodies

Antibodies used	Primary antibodies:<br>FLAG-M2 (F1804) Mouse mAb, Sigma-Aldrich<br>Anti-GPR110 C-termini antibody (SAB4501161), Sigma-Aldrich<br>beta-Tubulin (D3U1W) Mouse mAb, Cell Signalling Technology<br>GAPDH (14C10) Rabbit mAb, Cell Signalling Technology<br><br>Secondary antibodies:<br><br>IRDye® 800CW donkey anti-rabbit IgG #926-32213, Li-Cor Biosciences<br>IRDye® 680RD goat anti-mouse igG #926-68070, Li-Cor Biosciences
Validation	All primary antibodies were internally validated against empty vector loading controls in all western blots.<br><br>FLAG M2, beta-Tubulin and GAPDH used here are frequently used antibodies across a multitude of papers. The anti-GPR110 C-termini antibody was validated by sigma-aldrich at 1:500-1:1000 dilution, with bands at the appropriate mass in western blotting in “extracts from Jurkat/HuVEc/COLO cells using anti-GPR110 antibody... The lane on the right was treated with both anti-GPR110 antibody and the synthesized immunogen peptide.” No band was present when the synthesized peptide was present.

Eukaryotic cell lines

Policy information about cell lines and Sex and Gender in Research

Cell line source(s)	HEK293T (CRL-3216); ATCC<br>Expi293F™ Cells (A14527) ThermoFisher.<br>CRISPr HEKΔ6 line from Asuka Inoue.
Authentication	HEK293T and Expi293F cells were not authenticated. CRISPr HEKΔ6 line was validated by the lab of Asuka Inoue.
Mycoplasma contamination	Cell lines tested negative for mycoplasma.
Commonly misidentified lines<br>(See ICLAC register)	None used in this study
